# Reduced E-cadherin expression is correlated with poor prognosis in patients with bladder cancer: a systematic review and meta-analysis

**DOI:** 10.18632/oncotarget.19934

**Published:** 2017-08-04

**Authors:** Yongpeng Xie, Pin Li, Yu Gao, Liangyou Gu, Luyao Chen, Yang Fan, Fan Zhang, Xu Zhang

**Affiliations:** ^1^ Department of Urology, State Key Laboratory of Kidney Diseases, Chinese PLA General Hospital, Beijing, People’s Republic of China; ^2^ School of Medicine, Nankai University, Tianjin, People’s Republic of China; ^3^ Department of Urology, First Affiliated Hospital of Nanchang University, Nanchang, People’s Republic of China

**Keywords:** E-cadherin, prognosis, bladder cancer, biomarker, immunohistochemistry

## Abstract

The prognostic significance of E-cadherin expression in bladder cancer (BC) has been elevated for years, but published results remain controversial and inconsistent. We thus performed a systematic review and meta-analysis to determine the association between E-cadherin expression and BC prognosis. We systematically searched PubMed, Embase, Cochrane Library, and Web of Science databases to identify eligible studies published until March 2017. On the basis of our inclusion and exclusion criteria, a total of 2,089 patients from 19 studies were eligible for final analysis. Our results showed that reduced E-cadherin expression in BC was associated with poor overall survival (hazard ratio [HR] = 2.73, 95% CI: 1.74–4.27, *p* < 0.001), poor progression-free survival (HR = 6.39, 95% CI: 3.48–11.73, *p* < 0.001), and poor recurrence-free survival (HR = 2.48, 95% CI: 1.68–3.64, *p* < 0.001). Moreover, reduced E-cadherin expression was significantly correlated with pathological T stage (T2-4 vs. Ta-1: risk ratio [RR] = 2.14, 95% CI: 1.70–2.71), metastasis (yes vs. no: RR = 1.68, 95% CI: 1.17–2.40), grade (3 vs. 1/2: RR = 1.58, 95% CI: 1.29–1.93), and carcinoma *in situ* (yes vs. no: RR = 1.68, 95% CI: 1.09–2.58). This meta-analysis suggested that reduced E-cadherin expression was associated with poor prognosis and advanced clinicopathological characteristics and can serve as a useful biomarker for the clinical management of BC.

## INTRODUCTION

Bladder cancer (BC) is the most common malignancy of the urinary tract, with an estimated 76,960 new cases and 16,390 deaths in the United States of America in 2016 alone [1]. Clinically, BC is classified as non-muscle-invasive BC (NMIBC) and muscle-invasive BC (MIBC). At present, approximately 75% of BC cases are limited to the mucosa or submucosa at first diagnosis; however, about 50%–70% of NMIBC patients have tumor recurrence, and approximately 10%–30% cases progress to MIBC [2]. MIBC is highly aggressive and can rapidly progress and metastasize. Despite the improved therapeutic strategies available nowadays, most MIBC patients still eventually face death [3]. Hence, prediction models that can stratify patients who have an unfavorable prognosis and those who may benefit from early systematic therapy are greatly needed. Until date, tumor stage, grade, and metastasis are regarded as the major prognostic factors for BC. However, the currently used system seems unable to accurately predict the prognosis of BC patients with diverse and complicated tumor backgrounds [4]. Therefore, novel biomarkers that can identify patients at relatively greater risk when used alone, or in combination with other clinicopathological parameters, are required to precisely guide clinical decisions.

E-cadherin is a 120 kDa transmembrane, calcium-dependent cell adhesion protein that mediates cell-to-cell adhesion and maintains structural and functional integrity of epithelial tissues [5]. It also has pivotal barrier functions and maintains the polarity of epithelial cells [6]. Reduced or aberrant E-cadherin expression breaks cell-cell contacts, and thus, cells acquire the ability to migrate [7]. Consequently, aberrant E-cadherin expression promotes the infiltration and metastasis of cancer cells [8]. At present, several studies have reported that reduced E-cadherin expression is correlated with poor prognosis in several types of carcinomas [5, 7, 9–11]. However, the role of E-cadherin in the prognosis of BC remains controversial. Many studies have shown that reduced E-cadherin expression is associated with poor prognosis for BC patients [12–16], but some studies have suggested that there is no relationship between E-cadherin expression and prognosis in BC patients [17–20]. Moreover, numerous studies published in this field are small in size. Therefore, we conducted this systematic review and meta-analysis to quantitatively evaluate the prognostic and clinicopathological significance of E-cadherin expression in BC.

## RESULTS

### Search results

**Figure 1:**
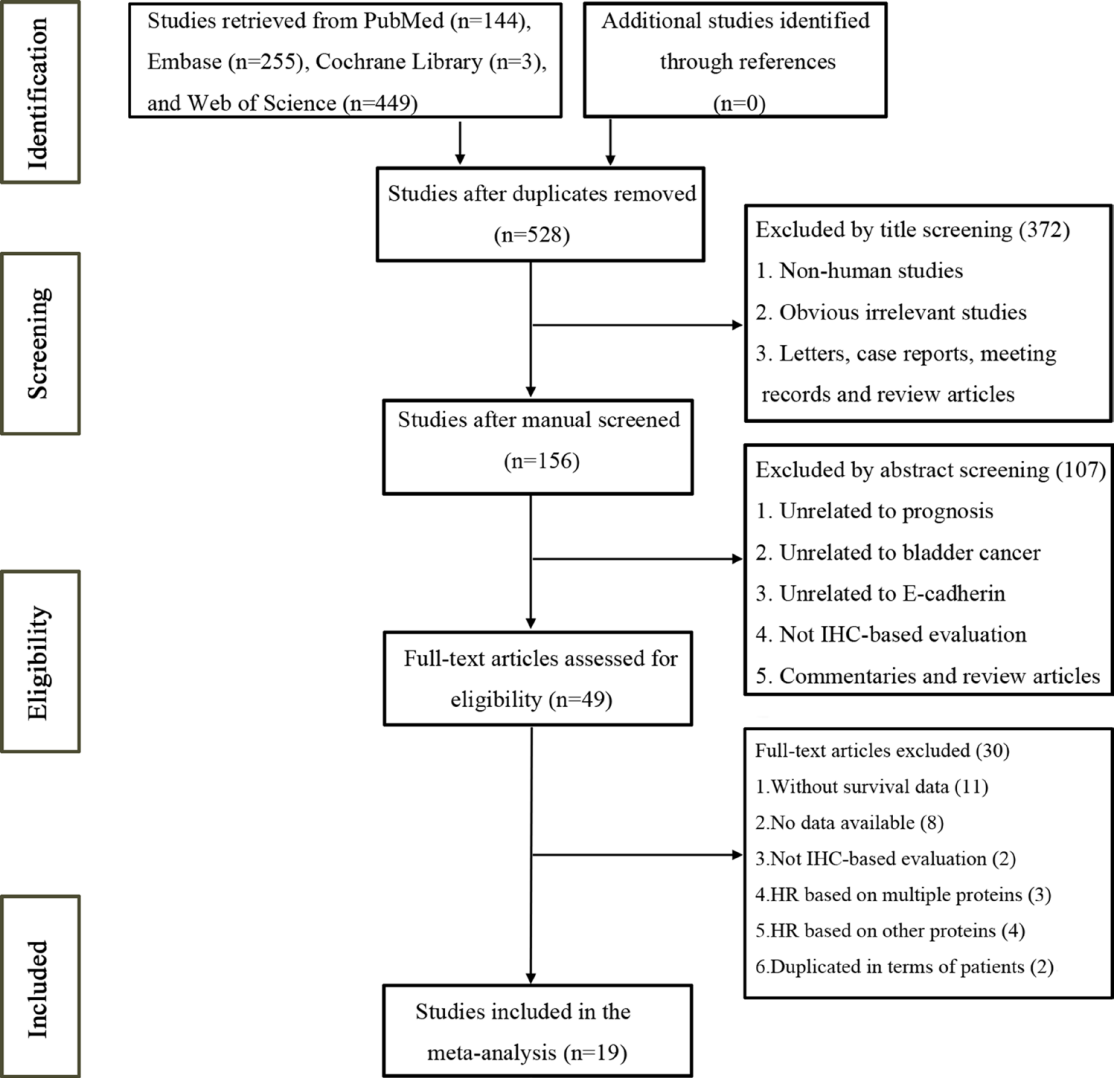
Flow chart of the study selection process

A total of 851 studies were potentially identified from the initial literature search of the databases [PubMed (*n* = 144), Embase (*n* = 255), Cochrane Library (*n* = 3), and Web of Science (*n* = 449)], and 528 of these studies were retained after duplicated studies were removed. By reviewing titles and abstracts, 479 studies were excluded because they were non-human studies, letters, case reports, meeting records, reviews, commentaries, and other obvious irrelevant studies. Additionally, to avoid heterogeneity caused by the method used to evaluate E-cadherin expression, studies without immunohistochemistry (IHC)-based evaluation were excluded. The remaining 49 studies were assessed in full text. Subsequently, 30 studies were excluded for various reasons such as without survival data, no data available, without IHC-based evaluation, HR based on multiple proteins, HR based on other proteins, and duplicated publication. Ultimately, a total of 19 studies with 2,089 patients were included in our meta-analysis [12–30]. A flowchart of the literature selection process is shown in Figure 1.

### Characteristics of studies

The fundamental features of the 19 studies are summarized in Table 1. All of these studies were retrospective observational cohort studies and were published between 1995 and 2017. Five studies evaluated patients from the United States, three from China, two from Germany, two from Japan, two from France, one from Italy, one from Sweden, one from Turkey, one from Spain, and one from the United Kingdom. For the prognostic value of reduced E-cadherin expression in BC, 12 studies investigated overall survival (OS), 9 studies investigated progression-free survival (PFS), and 6 studies investigated recurrence-free survival (RFS). Several clinicopathological data were reported in 12 studies (onset age in 4 studies, pathological T stage in 11 studies, metastasis in 4 studies, grade in 12 studies, and carcinoma *in situ* in 3 studies). All studies applied IHC staining to evaluate E-cadherin expression. Reduced E-cadherin expression was defined using different cut-off values among different studies; thus, we classified all the patients on the basis of their original studies (normal or reduced staining).

**Table 1:** Characteristics of eligible studies in the meta-analysis

Study	Year	Country	Study design	Pathological T stage	Case number	Sex (M/F)	Age (years)	Cut-off value	Reduced E-cadherin (%)	follow-up (months)	Survival analysis	HR estimated	Quality score*	Reference
Otto	2017	Germany	Cohort study	T1	226	173/53	Median 72	90%	73.5	Median 44	OS, PFS	Multivariable	8	17
Rosaria	2016	Italy	Cohort study	T1	92	80/12	Median 72.2	90%	50.0	13–170	OS	Multivariable	7	12
Breyer	2016	Germany	Cohort study	Ta	233	195/38	Median 70	IHC score^b^ < 3	43.9	> 66	PFS	Multivariable	7	13
Zhao	2014	China	Cohort study	T1-T3	121	90/31	Mean 67	50%	40.5	Median 72	PFS	Multivariable	7	18
Ding	2014	China	Cohort study	Ta-T2	49	31/18	40/9^a^ (> 60 y/≤ 60 y)	IHC score < 2	42.9	Median 40	RFS	Univariable	6	21
Mitra	2013	USA	Cohort study	Ta-T4	212	168/44	Median 58.9	NA	7.1	Median 13.2	OS	Multivariable	9	14
Muramaki	2012	Japan	Cohort study	Ta-T1	115	95/20	Median 69	90%	46.1	Median 34	RFS	Multivariable	8	22
Gudjonsson	2011	Sweden	Cohort study	Ta	52	40/12	Median 70	90%	53.1	Median 37.2	RFS	Univariable	6	19
Yu	2010	China	Cohort study	Ta-T4	120	87/33	56/64 (≥ 70 y/< 70 y)	10%	25.8	Median 30	OS, PFS	Multivariable	7	15
Fondrevelle	2009	France	Cohort study	Ta-T4	70	52/18	Median 69	90%	22.9	Median 30	OS, PFS	Multivariable	7	16
Fauceglia	2007	USA	Cohort study	T1	45	40/5	Mean 70	NA	24.4	Median 12	RFS, PFS	Multivariable	6	20
Kashibuchi	2007	Japan	Cohort study	Ta-T4	55	50/5	Median 62	IHC score <2	40.0	Median 29	OS	Multivariable	8	23
Erdemir	2007	Turkey	Cohort study	T1	52	36/16	Mean 64	90%	67.3	Mean 56.4	RFS	Multivariable	7	24
Baumgart	2007	USA	Cohort study	Ta-T4	299	231/68	Mean 67.1	IHC score <3	55.3	Mean 61.2	OS	Univariable	6	25
Shariat	2001	USA	Cohort study	Ta-T1	53	42/11	Median 66.8	90%	32.1	Median 131	OS, RFS, PFS	Univariable	8	26
Byrne	2001	USA	Cohort study	Ta-T4	77	60/17	Median 67	90%	76.6	Median 127.6	OS, PFS	Multivariable	8	27
Popov	2000	France	Cohort study	Ta-T4	111	92/19	Mean 65	30%	55.0	Median 36	OS	Multivariable	8	28
Muro	2000	Spain	Cohort study	Ta-T4	40	33/7	Median 69	20%	35.0	Median 24	OS, PFS	Multivariable	8	29
Syrigos	1995	UK	Cohort study	T1-T4	67	NA	NA	90%	76.1	> 60	OS	Univariable	6	30

IHC: immunohistochemistry; OS: overall survival; PFS: progression-free survival; RFS: recurrence-free survival; NA: not available.

^a^40 patients > 60 years, and other 9 patients ≤ 60 years.

^b^IHC score were evaluated by combining staining intensity and staining distribution.

*The quality of the included studies was evaluated using the Newcastle–Ottawa scale.

### Meta-analysis

**Figure 2:**
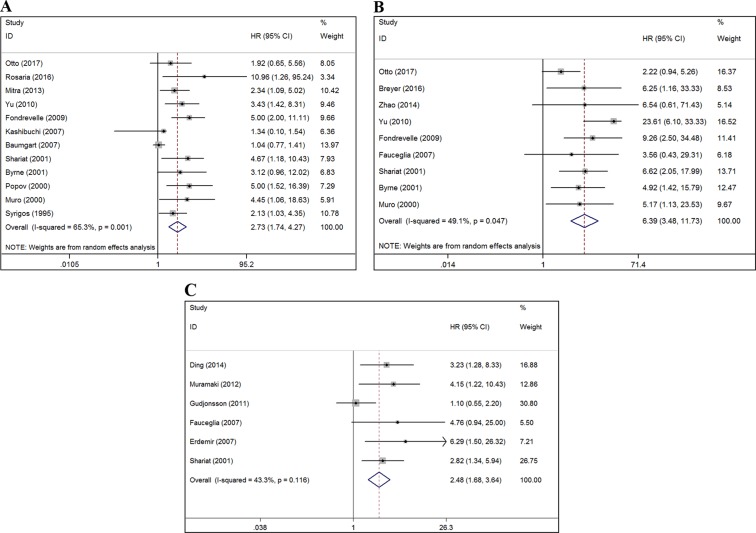
Forest plots of studies evaluating the correlation between E-cadherin expression and the prognostic outcomes of BC patients (**A**) Effect of reduced E-cadherin expression on OS. (**B**) Effect of reduced E-cadherin expression on PFS. (**C**) Effect of reduced E-cadherin expression on RFS. The red dash-line represents the pooled HR of the included studies. HR: hazard ratio; CI: confidence interval; OS: overall survival; PFS: progression-free survival; RFS: recurrence-free survival; BC: bladder cancer. HR > 1 implies unfavorable prognosis for patients with reduced E-cadherin expression.

This meta-analysis showed that reduced E-cadherin expression in BC patients predicted poor OS (a random-effect model, hazard ratio [HR] = 2.73, 95% confidence interval [CI]: 1.74–4.27, *p* < 0.001; *I^2^* = 65.3%, *p* = 0.001; Figure 2A), PFS (a random-effect model, HR = 6.39, 95% CI: 3.48–11.73, *p* < 0.001; *I^2^* = 49.1%, *p* = 0.047; Figure 2B), and RFS (a fixed-effect model, HR = 2.48, 95% CI: 1.68–3.64, *p* < 0.001; *I^2^* = 43.3%, *p* = 0.116; Figure 2C). To explore the source of heterogeneity among these studies, meta-regression analysis and subgroup analysis were performed on the basis of ethnicity, tumor extent, cut-off of staining, HR estimated method, and follow-up time (Table 2). For OS and PFS, meta-regression analysis revealed that cut-off of staining (*p* = 0.029) might have significant association with OS heterogeneity and ethnicity (*p* = 0.026) might be a significant contributor to the heterogeneity of PFS. For RFS, meta-regression analysis suggested that ethnicity, tumor extent, cut-off of staining, HR estimated method, and follow-up time were not significant contributors to heterogeneity (*p* = 0.178–0.862) (Table 2).

**Table 2:** Subgroup analysis of pooled HR for bladder cancer patients with reduced E-cadherin expression

Outcome	Subgroup	Studies	Pooled HR	95% CI	Model	Heterogeneity *I*^2^ (%)	Heterogeneity *p*-value	Meta-regression *p*-value
OS	Ethnicity							0.822
	Caucasian	10	2.84	1.70–4.74	random	69.2	0.001	
	Asian	2	2.59	1.24–5.41	fixed	24.2	0.251	
	Tumor extent							0.469
	NMIBC	3	3.44	1.67–7.07	fixed	21.2	0.281	
	NMIBC + MIBC	9	2.53	1.53–4.18	random	68.7	0.001	
	Cutoff of staining							0.029
	< 90%	3	4.02	2.13–7.59	fixed	0	0.873	
	≥ 90%	6	3.17	2.09–4.79	fixed	0	0.435	
	HR estimated							0.105
	univariable	3	1.92	0.84–4.38	random	78.1	0.010	
	multivariable	9	3.18	2.23–4.55	fixed	0	0.626	
	Follow up (month)							0.241
	< 40	6	3.28	2.19–4.92	fixed	0	0.542	
	≥ 40	6	2.29	1.21–4.31	random	66.8	0.010	
PFS	Ethnicity							0.026
	Caucasian	7	4.48	2.79–7.18	fixed	0	0.612	
	Asian	2	20.42	9.18–45.43	fixed	0	0.319	
	Tumor extent							0.143
	NMIBC	4	3.67	2.02–6.70	fixed	0	0.419	
	NMIBC+MIBC	5	10.94	6.29–19.03	fixed	33.6	0.197	
	Cutoff of staining							0.287
	< 90%	3	15.15	7.47–30.74	fixed	42.1	0.178	
	≥ 90%	4	4.31	2.52–7.38	fixed	29.1	0.238	
	HR estimated							0.964
	univariable	1	6.62	2.05–17.99	─	─	─	
	multivariable	8	6.31	3.11–12.83	random	55.5	0.028	
	Follow up (month)							0.111
	< 40	4	12.70	6.85–23.55	fixed	40.1	0.171	
	≥ 40	5	4.02	2.34–6.91	fixed	0	0.530	
RFS	Ethnicity							0.556
	Caucasian	4	2.57	1.17–5.66	random	58.1	0.067	
	Asian	2	3.60	1.78–7.29	fixed	0	0.730	
	Tumor extent							0.862
	NMIBC	5	2.78	1.43–5.40	random	52.6	0.077	
	NMIBC+MIBC	1	3.23	1.28–8.33	─	─	─	
	Cutoff of staining							0.613
	< 90%	0	─	─	─	─	─	
	≥ 90%	4	2.62	1.25–5.51	random	60.9	0.053	
	HR estimated							0.178
	univariable	3	2.07	1.04–4.14	random	57.3	0.096	
	multivariable	3	4.81	2.25–10.29	fixed	0	0.901	
	Follow up (month)							0.451
	< 40	3	2.42	0.85–6.89	random	64.4	0.060	
	≥ 40	3	3.31	1.93–5.67	fixed	0	0.622	

OS: overall survival; PFS: progression-free survival; RFS: recurrence-free survival; HR: hazard ratio; CI: confidence interval; NMIBC: non-muscle-invasive bladder cancer; MIBC: muscle-invasive bladder cancer.

The results of subgroup analysis are detailed in Table 2. With regard to ethnicity, reduced E-cadherin expression was associated with poor OS (HR = 2.84; 95% CI: 1.70–4.74; *p* < 0.001), PFS (HR = 4.48; 95% CI: 2.79–7.18; *p* < 0.001), and RFS (HR = 2.57; 95% CI: 1.17–5.66; p = 0.019) in Caucasian patients, and with poor OS (HR = 2.59; 95% CI: 1.24–5.41; *p* = 0.012), PFS (HR = 20.42; 95% CI: 9.18–45.43; *p* < 0.001), and RFS (HR = 3.60; 95% CI: 1.78–7.29; *p* < 0.001) in Asian patients. Regarding tumor extent, reduced E-cadherin expression predicted poor OS (HR = 3.44; 95% CI: 1.67–7.07; *p* = 0.001), PFS (HR = 3.67; 95% CI: 2.02–6.70; *p* = 0.003), and RFS (HR = 2.78; 95% CI: 1.43–5.40; *p* = 0.019) for NMIBC; and with poor OS (HR = 2.53; 95% CI: 1.53–4.18; *p* < 0.001), PFS (HR = 10.94; 95% CI: 6.29–19.03; *p* < 0.001), and RFS (HR = 3.23; 95% CI: 1.28–8.33; *p* = 0.013) for mixed BC (NMIBC + MIBC). For cut-off of staining, reduced E-cadherin expression was associated with poor OS (HR = 4.02; 95% CI: 2.13–7.59; *p* < 0.001) and PFS (HR = 15.15; 95% CI: 7.47–30.74; *p* < 0.001) when the cut-off value was less than 90%. Studies with a cut-off value greater than or equal to 90% revealed that reduced E-cadherin expression was correlated with poor OS (HR = 3.17; 95% CI: 2.09–4.79; *p* < 0.001), PFS (HR = 4.31; 95% CI: 2.52–7.38; *p* < 0.001), and RFS (HR = 2.62; 95% CI: 1.25–5.51; *p* = 0.011). With respect to HR estimated method, reduced E-cadherin expression was associated with poor OS (HR = 3.18; 95% CI: 2.23–4.55; *p* < 0.001), PFS (HR = 6.31; 95% CI: 3.11–12.83; *p* < 0.001), and RFS (HR = 4.81; 95% CI: 2.25–10.29; *p* < 0.001) under multivariable analyses and with poor PFS (HR = 6.62; 95% CI: 2.05–17.99; *p* = 0.002) and RFS (HR = 2.07; 95% CI: 1.04–4.14; *p* = 0.039) but not with poor OS (HR = 1.92; 95% CI: 0.84–4.38; *p* = 0.121) under univariable analyses. Moreover, reduced E-cadherin expression predicted poor OS (HR = 2.29; 95% CI: 1.21–4.31; *p* = 0.011), PFS (HR = 4.02; 95% CI: 2.34–6.91; *p* < 0.001), and RFS (HR = 3.31; 95% CI: 1.93–5.67; *p* < 0.001) in BC patients with a follow-up time greater than or equal to 40 months and poor OS (HR = 3.28; 95% CI: 2.19–4.92; *p* < 0.001) and PFS (HR = 12.70; 95% CI: 6.85–23.55; *p* < 0.001), but not poor RFS (HR = 2.42; 95% CI: 0.85–6.89; *p* = 0.098), in BC patients with a follow-up time less than 40 months.

In the comprehensive analyses of the significance of E-cadherin expression as a biomarker for BC, we explored the correlations between reduced E-cadherin expression and various clinicopathological features in patients. As illustrated in Table 3, reduced E-cadherin expression was significantly correlated with pathological T stage (T_2–4_ vs. T_a-1_: risk ratio [RR] = 2.14; 95% CI: 1.70–2.71; *p* < 0.001), metastasis (yes vs. no: RR = 1.68; 95% CI: 1.17–2.40; *p* = 0.004), Grade (3 vs. 1/2: RR = 1.58; 95% CI: 1.29–1.93; *p* < 0.001), and carcinoma *in situ* (yes vs. no: RR = 1.68; 95% CI: 1.09–2.58; *p* = 0.018). However, reduced E-cadherin expression was not significantly associated with onset age (> 70 vs. ≤ 70: RR = 1.17; 95% CI: 0.94–1.46; *p* = 0.153). There was significant inter-study heterogeneity in the analyses of pathological T stage and grade, but no significant heterogeneity was observed in any other parameters (Table 3).

**Table 3:** Meta-analysis of the association between reduced E-cadherin expression and clinicopathological features of bladder cancer

Variables	Studies	Pooled RR	95% CI	*P* Value	Model	Heterogeneity *I*^2^ (%)	Heterogeneity *p*-value
Age (>70 vs. ≤ 70)	4	1.17	0.94–1.46	0.153	fixed	4.8	0.369
pT stage (T_2–4_ vs. T_a–1_)	11	2.14	1.70–2.71	< 0.001	random	57.0	0.010
Metastasis^a^ (yes vs. no)	4	1.68	1.17–2.40	0.004	fixed	33.9	0.209
Grade (3 vs. 1/2)	12	1.58	1.29–1.93	< 0.001	random	71.8	< 0.001
CIS (yes vs. no)	3	1.68	1.09–2.58	0.018	fixed	45.1	0.162

^a^both lymph node and distant metastases; RR: relative ratio; CI: confidence interval; CIS: carcinoma *in situ*.

### Sensitivity analyses

**Figure 3:**
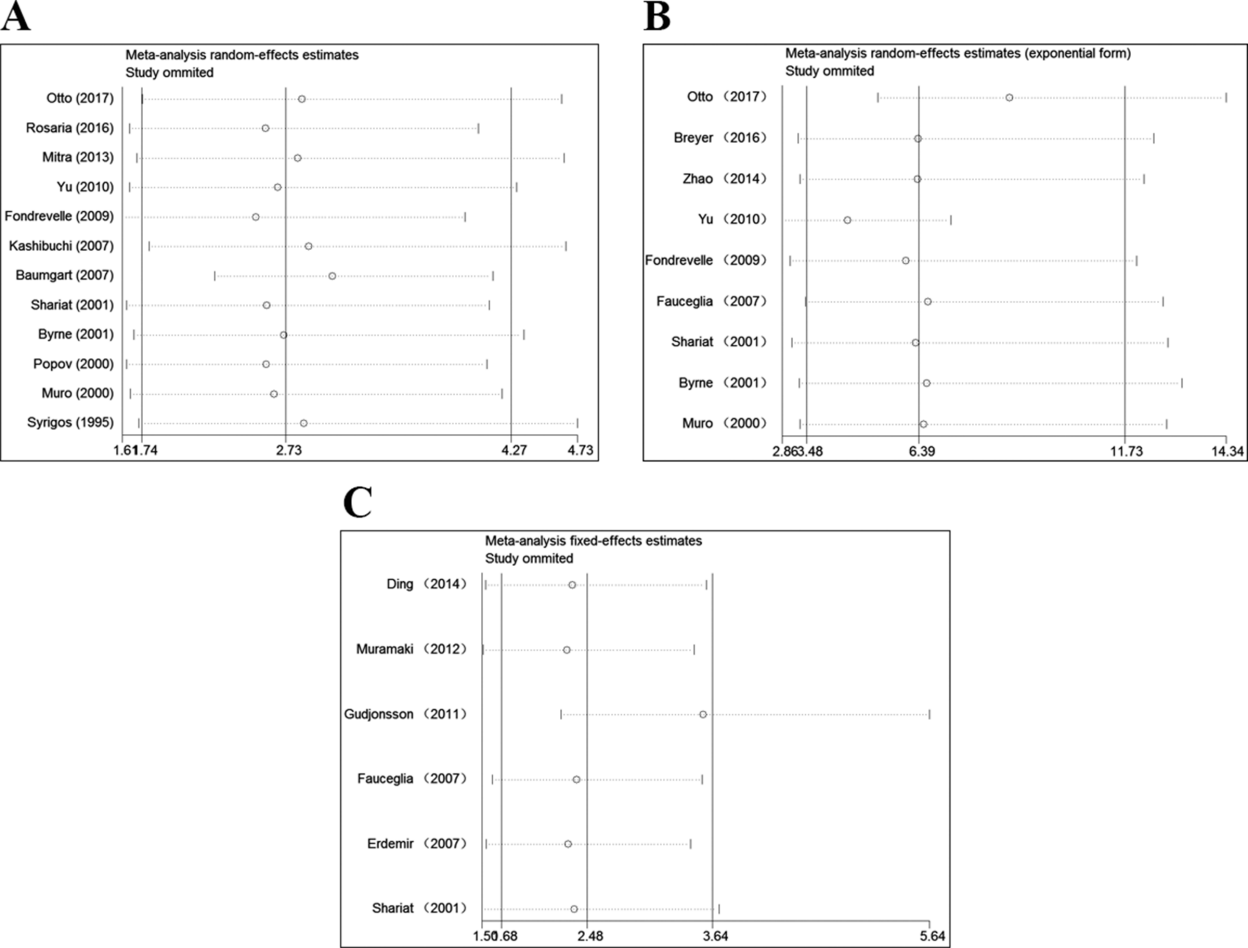
Sensitivity analysis for this meta-analysis (**A**) Sensitivity analysis for the reduced E-cadherin expression with OS. (**B**) Sensitivity analysis for the reduced E-cadherin expression with PFS. (**C**) Sensitivity analysis for the reduced E-cadherin expression with RFS. OS: overall survival; PFS: progression-free survival; RFS: recurrence-free survival.

To validate the stability of our results, we performed a sensitivity analysis by sequentially omitting individual studies. The pooled HR of OS, PFS, and RFS were not significantly influenced, thus indicating the robustness and reliability of our results (Figure 3).

### Publication bias

**Figure 4:**
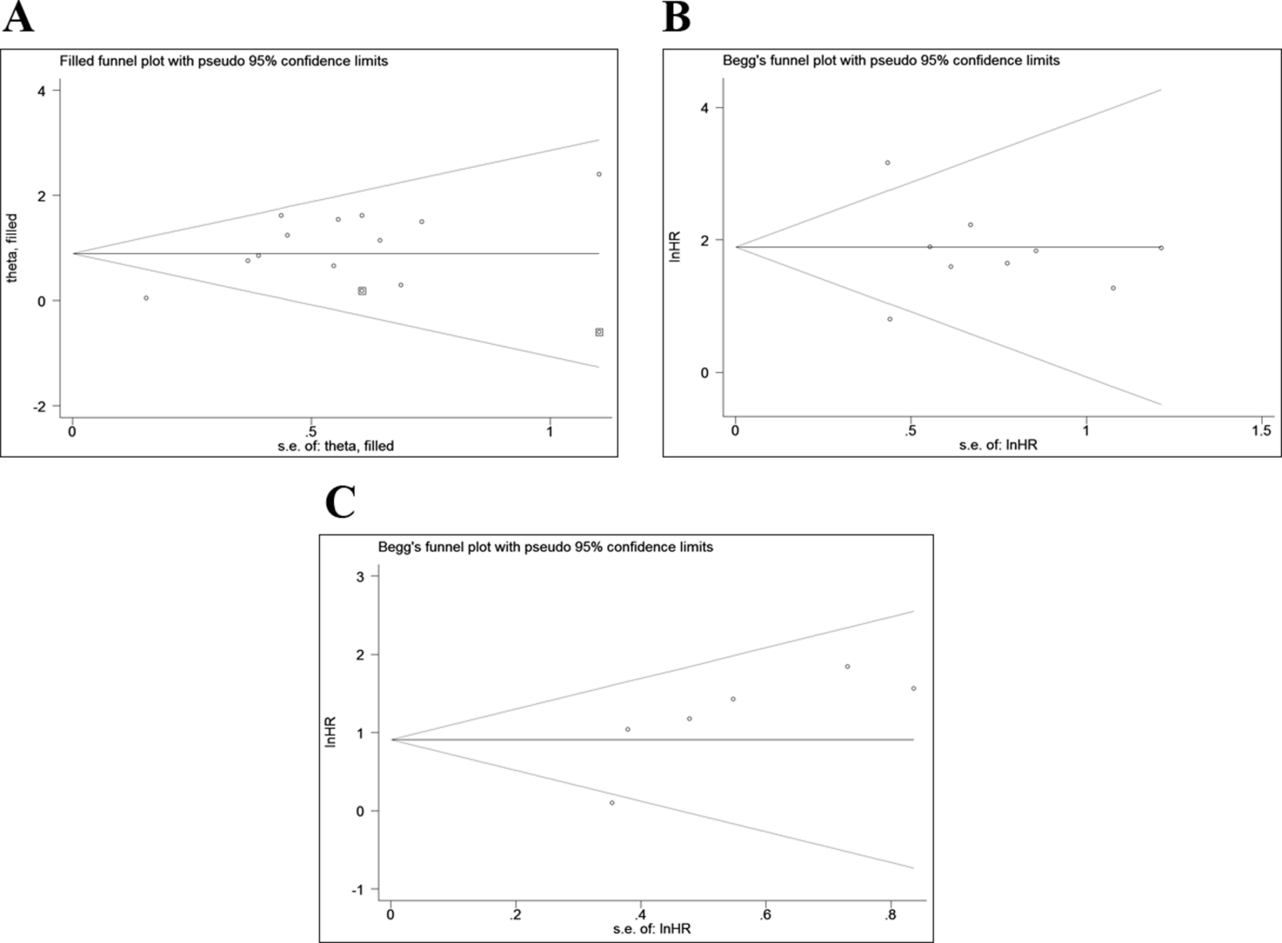
Funnel plots for the assessment of potential publication bias (**A**) Funnel plot of trim-and-fill analysis for the reduced E-cadherin expression with OS. (**B**) Funnel plot for the reduced E-cadherin expression with PFS. (**C**) Funnel plot for the reduced E-cadherin expression with RFS. OS: overall survival; PFS: progression-free survival; RFS: recurrence-free survival.

Begg’s test (*P* value) and Egger’s tests (*P* value, intercept with corresponding 95% CI), as well as funnel plots, were used to assess publication bias in this meta-analysis. As there were limited number of studies (*n* < 10) for PFS and RFS, publication bias evaluated by Begg’s and Egger’s test was not necessary. As illustrated in Figure 4, the funnel plots for PFS and RFS were symmetrical. However, both of these tests suggested that significant publication bias existed for OS (P_Begg’s_ = 0.451 and P_Egger’s_ = 0.001, intercept 2.63 with 95% CI: 1.48 to 3.79). Trim-and-fill analysis was performed and the results showed that after incorporating two additional studies, the funnel plots were symmetrical and that reduced E-cadherin expression was significantly associated with poor OS (corrected HR = 2.45; 95% CI: 1.62–3.71) (Figure 4).

## DISCUSSION

E-cadherin is an essential intercellular adhesion molecule that correlates with histogenesis and the stabilization and differentiation of epithelial cells [31]. It is generally known that down-regulation of E-cadherin expression is regarded as the most important hallmark of epithelial-to-mesenchymal transformation (EMT), which promotes the progression and metastases of many epithelium-derived carcinomas, including BC [32]. Over recent years, E-cadherin has been attracting increasing attention as a valuable prognostic predictor and potential therapeutic target for carcinomas. Several studies have confirmed that reduced E-cadherin expression is significantly correlated with the poor prognosis of gastric cancer, hepatocellular cancer, lung cancer, head and neck squamous cell carcinoma, and breast cancer [5, 7, 9, 10, 33]. However, the prognostic and clinicopathological roles of reduced E-cadherin expression remain inconsistent for BC. Thus, we performed this meta-analysis to resolve the remaining disagreement and provide valuable evidence on the association between reduced E-cadherin expression and BC prognosis. Additionally, to avoid the heterogeneity caused by the different methods used to evaluate E-cadherin expression, studies without IHC-based evaluation were excluded.

In this study, we focused exclusively on validating E-cadherin IHC-based expression and assessed the prognostic significance of reduced E-cadherin expression in BC patients. Our final analysis involved survival outcomes from 19 eligible studies including 2,089 BC patients. Our results showed that reduced E-cadherin expression significantly predicted unfavorable OS, PFS, and RFS. When we pooled survival data for OS and PFS, there was significant inter-study heterogeneity in our analyses. Consequently, meta-regression analysis and subgroup analysis were performed from five aspects. Meta-regression analysis revealed some significant sources of heterogeneity and suggested that cut-off of staining might have significant association with OS heterogeneity and that ethnicity might be a significant contributor to PFS heterogeneity. Through subgroup analysis, we revealed that reduced E-cadherin expression was significantly correlated with poor OS, PFS, and RFS, regardless of ethnicity, tumor extent, and cut-off of staining. In terms of HR estimated method, reduced E-cadherin expression was associated with poor OS, PFS, and RFS in multivariable analyses, which indicated that reduced E-cadherin might be an independent prognostic factor for survival outcome. Among patients with follow-up time less than 40 months, reduced E-cadherin expression was not significantly associated with poor RFS, even if patients with low-expression of E-cadherin presented with a relatively unfavorable RFS. The absence of a significant correlation in this situation was possibly attributed to the relatively limited number of studies in the subgroups.

Our results also suggested that the down-regulation of E-cadherin expression was associated with a higher pathological T stage, positive metastasis, grade, and carcinoma *in situ*. The biological mechanism of E-cadherin can partially explain its prognostic and clinicopathological value for BC patients. E-cadherin routinely plays an inhibitory effect on EMT. Reduced E-cadherin expression may therefore induce EMT, which increases tumor cell mesenchymal characteristics and subsequently promotes cell motility and invasive properties [34, 35]. This process accelerates the development and progression of malignant tumors. Furthermore, the low-expression of E-cadherin is very closed associated with the chemoresistance and radioresistance of tumor cells and induces tumor cells to exhibit obvious properties of cancer stem cells [36, 37].

To the best of our knowledge, this study is the first systematic and comprehensive analysis to investigate the associations between E-cadherin expression and prognostic and clinicopathological value in BC patients, although some limitations should be pointed out. First, most of the included studies were retrospective studies that might render our conclusions less reliable. Second, all of the included studies evaluated E-cadherin expression via IHC, although different primary antibody sources and antibody dilution ratios could have resulted in differences in terms of IHC sensitivity. Third, the criteria to define normal or reduced expression of E-cadherin were not uniform across different studies, which may potentially lead to heterogeneity. Thus, a more uniform scoring criteria should be defined in the future. Fourth, relatively limited studies were extracted for some subgroup analyses, which might inevitably increase the risk of random error and contribute to premature results. With more large-scale prospective studies published in the future, an update is necessary to render a more convincing result. Finally, studies with statistically significant results are potentially more likely to be submitted and published, than those with non-significant results, which could generate publication bias [38].

## MATERIALS AND METHODS

### Search strategy

This meta-analysis was performed in accordance with the guidelines of the Preferred Reporting Items for Systematic Reviews and Meta-Analyses (PRISMA) [39].

A systematic literature search was performed in the electronic databases PubMed, Embase, Cochrane Library, and Web of Science on March 1, 2017, using the following search strategy: (“E-cadherin” or “E-CAD” or “cadherin-1” or “CDH1”) and (“bladder cancer” or “bladder tumor” or “bladder carcinoma” or “bladder neoplasm” or “urothelial cancer” or “urinary tract cancer”) and (“prognosis” or “prognostic” or “survival” or “outcome” or “mortality”). Additionally, we manually searched the references section of all eligible literature.

### Selection criteria

Studies were included on the basis of the following criteria: (1) studies that reported the association between E-cadherin expression and its prognostic significance in BC; (2) studies that assessed E-cadherin protein expression using IHC; and (3) studies that described survival outcomes (OS, PFS, or RFS) with HR and 95% CI. Exclusion criteria were as follows: (1) non-English papers; (2) non-human studies; (3) letters, case reports, meeting records, commentaries, or review articles; (4) studies that investigated the survival outcomes of BC based on multiple proteins; (5) studies that did not evaluate E-cadherin protein expression, clinical parameters, and survival outcome; and (6) studies that were unable to provide sufficient data for obtaining HR and 95% CI values. All evaluations were independently conducted by three investigators to ensure the accurate inclusion of studies. For duplicate data, only studies with relatively more details and larger sample sizes were retrieved.

### Data extraction

Three individual researchers independently extracted data from the included studies using a predefined form. Discrepancies in data extraction were resolved through negotiation and consultation. The following relevant data were extracted: publication data including author names and the year in which the study was conducted; origin of the studied population; study design; pathological T stage; sample size; sex; patient’s age; cut-off value; follow-up time; and effect estimates, namely, HR of E-cadherin expression for OS, PFS, or RFS, as well as their 95% CI (Table 1).

### Quality assessment

The Newcastle–Ottawa scale, which was recommended by the Cochrane Non-Randomized Studies Methods Working Group [40], was used to assess the quality of the included studies. The assessment, with a score ranging from 0 to 9, included three perspectives: selection, comparability, and outcomes. Studies with scores higher than 6 were considered to be of high quality. To assure the quality of this meta-analysis, only high-quality studies included.

### Statistical analysis

Pooled HR and RR with 95% CI were used to evaluate the effect of reduced E-cadherin expression on the prognosis and clinicopathological features of BC, respectively. An observed HR > 1 implied a relatively worse prognosis for the group with reduced E-cadherin expression. An observed RR > 1 indicated relatively more advanced clinicopathological features for patients with reduced E-cadherin expression. The heterogeneity of the studies was assessed using Cochran’s *Q* test and Higgins I-squared statistic. When significant heterogeneity was observed (*I^2^* > 50% or *p* < 0.05), a random-effect model was used; otherwise, a fixed-effect model was chosen (*I^2^* < 50% and *p* > 0.05). For additional analysis, subgroup analyses were performed to investigate the association between reduced E-cadherin expression and BC prognosis on the basis of ethnicity, tumor extent, cut-off of staining, HR estimated, and follow-up time. Meta-regression analysis was used to explore the source of inter-study heterogeneity. Potential publication bias was assessed using Begg’s and Egger’s tests. We also performed a sensitivity analysis by sequential omitting individual studies to evaluate the robustness of pooled results. All statistical analyses were performed using Stata 12.0 software (Stata Corporation, College Station, TX, USA), and a two-sided *p* < 0.05 was considered statistically significant.

## CONCLUSIONS

In conclusion, despite these limitations, our meta-analysis suggested the prognostic and clinicopathological significance of E-cadherin expression in patients with BC. Our results revealed that reduced E-cadherin expression predicted poor prognosis and advanced clinicopathological characteristics, which could potentially serve as a risk stratification biomarker, and even a valuable therapeutic target, for BC patients. However, more large-scale prospective studies using uniform criteria and long-term follow-up are required to confirm our findings in the future.
