# Correction: Reduced E-cadherin expression is correlated with poor prognosis in patients with bladder cancer: a systematic review and meta-analysis

**DOI:** 10.18632/oncotarget.26865

**Published:** 2019-04-05

**Authors:** Yongpeng Xie, Pin Li, Yu Gao, Liangyou Gu, Luyao Chen, Yang Fan, Fan Zhang, Xu Zhang

**Affiliations:** ^1^ Department of Urology, State Key Laboratory of Kidney Diseases, Chinese PLA General Hospital, Beijing, People’s Republic of China; ^2^ School of Medicine, Nankai University, Tianjin, People’s Republic of China; ^3^ Department of Urology, First Affiliated Hospital of Nanchang University, Nanchang, People’s Republic of China

**This article has been corrected:** The correct author information is given below:

**Pin Li**^2,1,*^ **and Xu Zhang**^**1,2**^

Original article: Oncotarget. 2017; 8:62489-62499. https://doi.org/10.18632/oncotarget.19934

